# The significance of a ‘T shaped’ incision for resection of giant mediastinal tumors: a case report

**DOI:** 10.1093/jscr/rjae354

**Published:** 2024-05-30

**Authors:** Manjinder Kaur Pannu, Jonas Peter Ehrsam, Othmar Markus Schöb, Ilhan Inci

**Affiliations:** Department of Basic and Clinical Sciences, University of Nicosia Medical School, Nicholas St 93, CY-2408, Nicosia, Cyprus; Klinik Hirslanden Zürich, Thoracic Surgery Clinic, Witellikerstrasse 40, 8032, Zürich, Switzerland; Klinik Hirslanden Zürich, Thoracic Surgery Clinic, Witellikerstrasse 40, 8032, Zürich, Switzerland; Department of Basic and Clinical Sciences, University of Nicosia Medical School, Nicholas St 93, CY-2408, Nicosia, Cyprus; Klinik Hirslanden Zürich, Thoracic Surgery Clinic, Witellikerstrasse 40, 8032, Zürich, Switzerland

**Keywords:** liposarcoma, surgical oncology, surgical wound, thoracic surgery

## Abstract

A 37-year-old male, with a 5-year history of liposarcoma of the right thigh, was incidentally diagnosed with two huge thoracic metastases following a fall. One of these masses, measuring 22 cm, was located in the right chest apex, adjacent to a second 20 cm mass situated in the anterior mediastinum, partially invading the left chest. The patient underwent surgical intervention for mass resection that commenced with a hemi-clamshell incision, but was then extended by completing the lower median sternotomy in order to create a T shaped incision. This type of incision provides ample access for large mediastinal tumors that extensively extend into one side of the thoracic cavity, encompass the anterior mediastinum, and partially reach into the opposite cavity. It enhances visualization, facilitates access to vital organs, allows for precise surgical maneuvers, minimizes the risk of inadvertent tissue damage, and enables thorough oncological resection.

## Introduction

Giant mediastinal tumors (GMTs) are extremely uncommon with varying definitions in the literature, described either as occupying at least half of the hemithorax or having a diameter exceeding 10 cm [1] or 20 cm [2]. Germ cell tumors (33%), liposarcoma (10%), and thymoma (10%) are the primary causes of GMTs [1].

**Figure 1:**
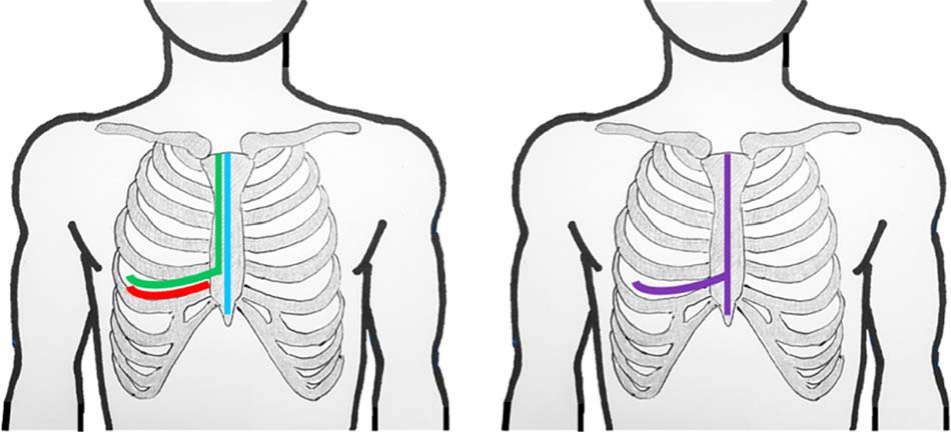
Schematic lines of incision for anterolateral thoracotomy (red); hemiclamshell (green); sternotomy (blue); T-incision (violet).

In the anterior mediastinum, common approaches for GMT resection include anterolateral thoracotomy and median sternotomy. For posterior mediastinal GMTs, posterolateral thoracotomy is preferred, and for those situated more deeply in the chest cavity, hemi-clamshell incisions are used [1, 3]. A clamshell incision is the traditional approach when the tumor extends into both hemithoraces [3]. The literature rarely reports on the advantages of a T (⊣) shaped incision, involving a complete sternotomy combined with anterolateral thoracotomy (Fig. 1).

This case report details a unique scenario involving two adjacent GMTs, in the anterior mediastinum and the right upper chest, successfully resected using such a ⊣ shaped incision.

## Case report

A 37-year-old male arrived at our hospital as a surgical referral. In 2018, he underwent surgery for liposarcoma in the right thigh’s musculus sartorius, preceded by neoadjuvant radiotherapy. In 2021, a punch biopsy confirmed a solitary sacral metastasis of the same liposarcoma, leading to additional radiotherapy. Despite being asymptomatic for two years, in 2023, a fall caused persistent dyspnea and retrosternal pain, leading to a hospital visit.

**Figure 2:**
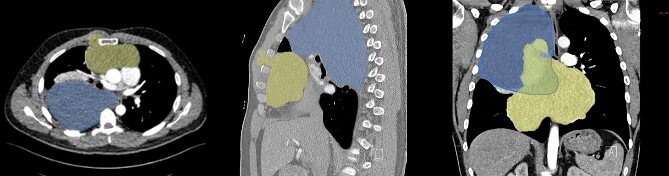
CT scan of tumor dimensions.

During examination, physicians observed a parasternal swelling, prompting computed tomography (CT) scan (Fig. 2) due to the patient’s extensive medical history and new symptoms. The scan revealed a large 22 cm hypodense lesion in the upper right thorax, partially compressing the superior vena cava and vena subclavia dextra. The lesion showed extensive contact with the trachea and esophagus without signs of infiltration, along with adjacent atelectasis of the upper and middle lobes. Another mass, 20 cm in size, was discovered in the anterior mediastinum, extending into the sternum and musculus pectoralis major. These tumors exhibited no postcontrast enhancement or calcification. Strong suspicion of secondary or metastatic liposarcoma prompted scheduling surgery after presenting the case at a multidisciplinary meeting. Veno–veno extracorporeal membrane oxygenation was planned on standby due to the inability of preoperative imaging to rule out central vessel infiltration.

After an interdisciplinary meeting, surgical resection without adjuvant therapy was decided.

**Figure 3:**
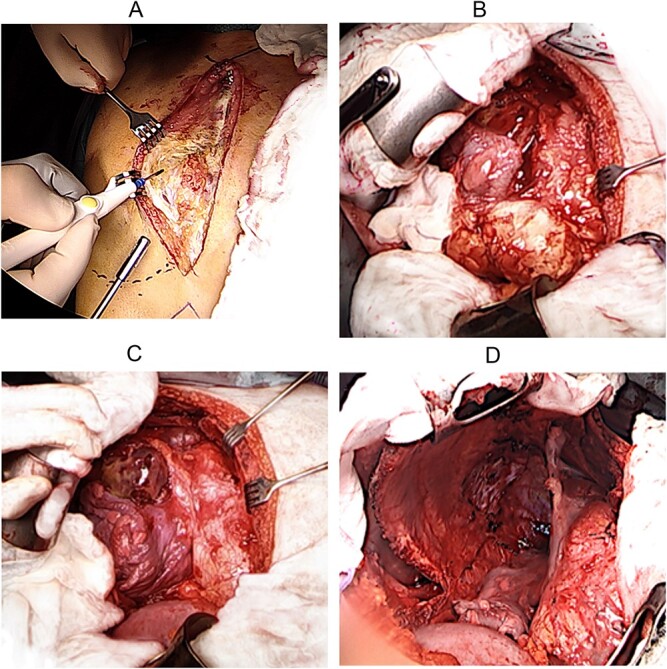
Perioperative situs.

The surgical procedure began with a right-sided hemi-clamshell through the fourth intercostal space, initiating the pectoral resection of the transthoracic part of the tumor in the anterior mediastinum with secure margins (Fig. 3A). However, the strongly adherent tumor posed challenges in opening the hemi-clamshell window and significantly restricted exposure to the left mediastinal part. Consequently, a decision was made to expand the incision by completing the sternotomy caudally, thus creating a ⊣ shaped incision (Fig. 3B).

Following this modification, a radical en bloc resection of the mass within its capsule in the anterior mediastinum, inclusive of the thymus, from the right and left recessus costodiaphragmalis, the pericardium, the superior vena cava, anonyma vein, ascending aorta, pulmonary trunk, and internal mammary vessels under the protection of bilateral phrenic nerves, became feasible (Fig. 3C). Subsequently, the second tumor in the upper right chest cavity was successfully detached en bloc within its capsule from the lung, trachea, chest cavity, with ligation of the in- and outflow branches of the subclavian vessels. In the posterior mediastinum, detachment from the esophagus was carried out.

The entire operation lasted 2 h and 55 min and, remarkably, despite the extensive incision and large procedure, intraoperative blood loss totaled only 600 ml. The patient was discharged from the hospital on Day 10, without complications.

**Figure 4:**
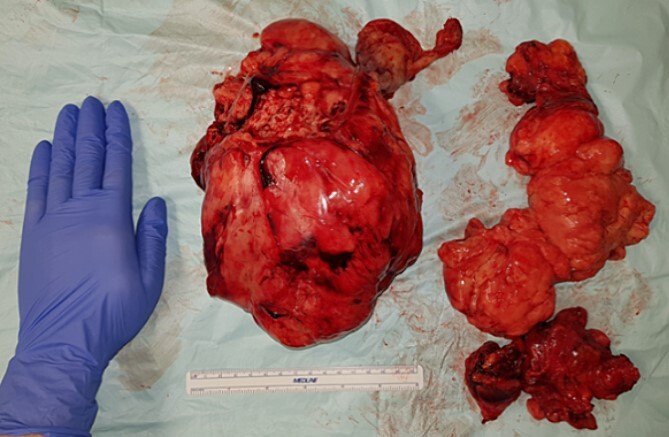
The two en bloc removed tumor specimens.

The two resected tumors measured 22 × 18 × 6 cm (Fig. 4) and 20 × 15 × 6 cm, weighing 1400 g and 508 g, respectively. Both tumors removed were enclosed by a delicate membrane, and subsequently showed negative margins on final pathology, in addition to a myxoid consistency indicating their mesenchymal origin. The tumors predominantly consisted of uniform, small spindle to stellate-shaped cells. Immunohistochemical analysis showed a focal proliferation index of up to 30%. The tumors tested negative for pancytokeratin EDMA, MDM2, Muc4, MyoD1, Myogenin, CD34, and ALK1. Fluorescence in situ hybridization (FISH) analysis revealed a clear rearrangement of the DDIT3 gene, confirming the diagnosis of metastasis from the previously known myxoid liposarcoma.

At the 6-month full-body magnetic resonance imaging (MRI) follow-up, no disease recurrence was detected.

## Discussion

The combined sternotomy and hemi-clamshell approach, termed the ‘⊣ shaped’ incision [4], should be considered where a GMT extends from the anterior thorax region into one hemithorax while also partially involving the other hemithorax.

In our case, a GMT was situated in the right chest apex, neighbored by a second GMT in the anterior mediastinum, partially invading the left chest. The substantial size of the two neighboring tumors and our patient’s history of prior metastases made complete surgical excision imperative. Furthermore, it was vital to confirm during the surgical procedure that there was no spread of the tumors into adjacent tissues.

The advantages of the ⊣ shaped incision are significant. First, it can be adapted from a sternotomy, anterolateral thoracotomy, or a clamshell incision (Fig. 1), providing flexibility when uncertainty initially exists about the need for a ⊣ shaped incision. Second, this approach offers exceptional visibility into one thoracic cavity, and additionally covers the anterior mediastinum and extends, at least partially, into the contralateral thoracic cavity.

In addition to underscoring the distinctive surgical approach highlighted in this report, it is essential to emphasize the rarity of the two secondary liposarcoma GMTs in our case. Although there have been recorded cases of liposarcoma metastasizing to the mediastinum [5, 6], such occurrences are reported to be even less common than primary mediastinal liposarcomas [7]. The latter of which is described as exceedingly rare, comprising <1% of all cases [8]. A comprehensive understanding of the specific patterns of liposarcoma metastases has not been conclusively established.

In conclusion, a ⊣ shaped incision should be evaluated by surgeons when confronted with complex thoracic surgeries involving GMTs. The compelling benefits encompassing enhanced exposure, complete oncological resection, and decreased risks of intra- and postoperative complications, collectively advocate for the adoption of this incision type where indicated.

## Data Availability

The datasets used are available from the corresponding author on reasonable request.

## References

[1] Shi X , Liu X, Dong X, et al. Trends, symptoms, and outcomes of resectable giant mediastinal tumors. Front Oncol 2022;12:820720. 10.3389/fonc.2022.820720. 35186755 PMC8854276

[2] Ataya J , Nahle A, Hamdar H, et al. Mediastinal liposarcoma: a case report and review of the literature. J Med Case Reports 2023;17:372. 10.1186/s13256-023-04121-7. PMC10469449 37649065

[3] Bains S , Ginsberg J, Jones G 2nd, et al. The clamshell incision: an improved approach to bilateral pulmonary and mediastinal tumor. Ann Thorac Surg 1994;58:30–3 discussion 33. 10.1016/0003-4975(94)91067-7. 8037555

[4] Huang W , Jiang N. Resection of giant mediastinal liposarcoma via ‘⊣shape’ incision. J Surg Case Rep 2017;2017:rjw 219. 10.1093/jscr/rjw219. PMC5204133 28044001

[5] Liu J , Song Z, Liu R, et al. Relapsed pleomorphic liposarcoma with mediastinal metastasis: a case report and review of the literature. Zhong guo Fei Ai Za Zhi 2017;20:361–5 Chinese. 10.3779/j.issn.1009-3419.2017.05.10. PMC5973061 28532545

[6] Inuganti RV , Bala SG, Bharathi KY. Metastatic myxoid liposarcoma of lung and mediastinum diagnosed by fine needle aspiration. J Cytol 2011;28:33–5. 10.4103/0970-9371.76948. 21552406 PMC3083533

[7] Suster D , Suster S. Liposarcomas of the mediastinum. Mediastinum 2020;4:27. 10.21037/med-20-42. 35118295 PMC8794306

[8] Bagaria P , Gabriel E, Mann N. Multiply recurrent retroperitoneal liposarcoma. J Surg Oncol 2018;117:62–8. 10.1002/jso.24929. 29266232

